# ‘Menstruation means impurity’: multilevel interventions are needed to break the menstrual taboo in Nepal

**DOI:** 10.1186/s12905-021-01231-6

**Published:** 2021-02-28

**Authors:** Subash Thapa, Arja R. Aro

**Affiliations:** 1 Research Unit of General Practice, Department of Public Health, University of Southern Denmark, Odense, Denmark; 2 Unit for Health Promotion Research, University of Southern Denmark, Esbjerg, Denmark; 3 EduRes Consulting Ltd, Veikkola, Finland

**Keywords:** Community mobilization, Menstrual health interventions, Menstruation management interventions, Menstrual stigma, Menstrual taboo, Multilevel interventions, Women’s health, Nepal

## Abstract

**Background:**

During their menstrual period, women are generally considered impure in Nepal; in the rural areas of the western part of the country, they are even banished to stay in sheds (called chhaupadi) during this time, which increases their vulnerability to a variety of health consequences. There is lack of clarity regarding the effectiveness of interventions that have been implemented to address menstrual taboo and improve menstrual hygiene and practices in Nepal (e.g., public awareness, community sensitization and legislation). In this paper, we discuss why menstruation management interventions, particularly those implemented to change the menstrual taboo might not work, and the opinions and experiences regarding the implementation of such interventions.

**Main text:**

Anecdotal reports from the field and empirical studies suggest that interventions to address menstrual taboos have only been effective for short durations of time due to several reasons. First, local community stakeholders have been reluctant to take actions to abandon retrogressive menstrual practices in rural areas. Second, women who have stopped practising chhaupadi have faced stigma (e.g., fear of exclusion) and discrimination (e.g., blaming, physical and verbal abuse). Third, contextual factors, such as poverty and illiteracy, limit the effectiveness of such interventions. Fourth, community sensitization activities against chhaupadi have faced resistance from community leaders and traditional healers. Fifth, the law prohibiting chhaupadi has also faced implementation problems, including poor filing of complaints.

**Conclusion:**

Multilevel, multisectoral interventions could be more effective than single-component interventions in breaking the prevailing menstrual taboo and in improving menstrual health and hygiene practices among young girls and women in the rural areas of Nepal. Moreover, interventions that have an active community mobilization component could be effective within local contexts and cultural groups.

## Background

In Nepal, the social meaning of menstruation is impurity, and during their menstrual period, women are not allowed to stay in the family [1]. In the rural areas of the western part of Nepal, women are even banished to sheds during their menstrual period, which is a tradition known as chhaupadi. Rooted in the beliefs of Hinduism, menstrual restrictions (e.g., restrictions regarding attending school and accessing health care, food and drink) including chhaupadi are generally considered a purity practice by the local people; as such, God’s rules are obeyed, and potential misfortunes are prevented [2]. This practice, however, challenges fundamental human rights and central ethical principles. For instance, the practice increases women’s vulnerability to a variety of health consequences, such as illness due to exposure to cold, heath problems due to sleeping and living in unhygienic conditions, animal attacks, snake bites, rape and sexual assaults, and poor access to health care [1, 3].

A number of interventions to address menstrual taboo and improve overall menstrual health and hygiene have been implemented in Nepal over the last decade. Such initiatives are generally referred to as menstruation management interventions, and their aim is to ensure that women and girls can manage their periods in a way that is not only healthy but also enables their full participation in school, work, and other activities [4]. Examples of such interventions include raising awareness and advocacy strategies using street dramas, posters, booklets, and radio campaigns; developing national guidelines for menstrual health management and education on menstrual health and hygiene (e.g., school curricula on menarche, healthy menstrual practices, the use of menstrual aids such as sanitary napkins, and the management of menstrual disorders); and the construction of toilet facilities in schools [5, 6].

In relation to chhaupadi, an elimination campaign has been initiated that mainly includes community sensitization through raising awareness and conducting menstrual-shed destruction activities [7]. In addition, a criminal code law against chhaupadi was enacted on 9 August 2017, and according to this law, any family member who forces a woman to practice chhaupadi can be punished with a jail sentence of 3 months and/or a fine of approximately $30 [8]. The law further prohibits discrimination against and the inhuman treatment of menstruating women, as well as segregating them as untouchables [8].

The interventions are believed to change the perceptions of women and community members, and the number of women practising chhaupadi is believed to have been declining over the years; however, the rate of change is rather slow [9]. It is also believed that the effectiveness of such interventions is only documented on paper and not in real-world practice, i.e., that the vast majority of families and women are still continuing to practice chhaupadi or other menstrual restrictions [5, 7]. A quantitative study conducted in 2019 reported that menstruation management interventions have not been effective in improving knowledge and practices regarding menstrual health, and that women in rural areas, in particular, have poor menstrual health outcomes [10]. To date, there is insufficient evidence on the effectiveness of menstrual management interventions in terms of positive menstrual health outcomes or how such interventions should be implemented in different cultural contexts in Nepal [4].

Based on the review of available gray and peer-reviewed evidence, in the present paper, we discuss why menstruation management interventions, particularly those implemented to change the menstrual taboo, including chhaupadi, might not work, and the opinions and experiences regarding the implementation of such interventions. We did not follow a systematic process but based the review on general topic-related keyword search (e.g., “chhaupadi”, “menstrual health interventions”, “menstruation management intervention”, “menstruation management programme”, “menstrual restrictions”, “menstrual stigma”, “menstrual taboo”) on PubMed, Google Scholar and official websites of non-governmental organizations implementing programmes and interventions to address menstrual taboo in Nepal. Altogether seven programme or news reports [5–7, 11–14] and six peer-reviewed papers [1, 2, 9, 10, 15, 16] explaining menstrual taboo and/or menstrual management interventions were included in the review. We also looked for additional relevant programme reports and peer-reviewed papers [4, 8, 17–22] to consider implementation perspectives on how the effectiveness of the interventions can be enhanced.

## Main text

### Why interventions might not work

Despite several interventions to address menstrual taboo (e.g., awareness raising, community sensitization, and legislation), the majority of young girls and women are still forced to follow different traditional menstrual practices (e.g., chhaupadi, untouchability and restrictions) [15]. Anecdotal reports from the field [7, 11, 14] and empirical studies [9, 10] have suggested that these interventions have only been effective for a short duration of time for several reasons. For instance, a baseline programme evaluation report published in 2015 suggested that community stakeholders were reluctant to participate in interventions aimed at abolishing such practices, although most of them initially showed their interest and support [7]. Moreover, rumors that blame the women who stop practising menstrual traditions for negative consequences in the community, such as sudden illnesses, accidents or deaths in families or neighbourhoods, circulate within communities [1]. Incidences of physical and verbal abuse towards such women and the exclusion of the families that endorse abandoning the tradition have also been reported in newspapers [14]. As the menstrual taboo has been internalized and perceived as the routine aspect of life by so many women, they fear that not following this tradition may lead to other consequences in their lives, such as marriage rejections or abandonment after the marriage [1].

Traditional beliefs and practices are more pronounced in the rural communities of the western part of Nepal than elsewhere in the country [23]. In these regions, poverty, illiteracy and the practice of traditional healing are contextual factors that could partly limit the effect of menstrual management interventions [7, 9]. For instance, even if people gradually change their attitudes and are ready to accept menstruating women staying inside the house in a separate room with a few menstrual restrictions (e.g., restrictions regarding entering the kitchen), a majority of the households in the western region of Nepal do not have an extra room for women to use during their periods due to widespread poverty; thus, women from these households are forced to stay in sheds [9].

The menstrual taboo is generally believed to be rooted in Hinduism and is often characterized as social control over a woman’s body and behaviour that is interwoven in complex ways with religious beliefs [2, 16]. In this view, religious leaders and traditional healers often raise their voices against community activities that are aimed towards changing the menstrual taboo. For instance, the baseline programme evaluation report of 2015 revealed that a majority of male community leaders did not perceive chhaupadi as being a relevant problem of the women and girls in their communities; in particular, male traditional healers were highly reluctant to change discriminatory practices during menstruation [7]. Moreover, chhaupadi differs from some other forms of violence against girls and women in that women are not only the victims but also involved in perpetration; however, the current interventions do not target women as perpetrators at all [20]. There is, therefore, a high need to incorporate pertinent strategies to involve, convince and mobilize local women and male community leaders who exert some level of control over and influence on the prevailing socio-economic and cultural structure of families and communities. Without acceptance and enforcement by these groups, the interventions aimed at breaking the menstrual taboo are likely to face resistance by communities.

Uma (2019) noted that the law prohibiting chhaupadi has a glaring loophole in that ‘it only prohibits persons from forcing a woman to follow the custom and does not prohibit women from following the practice voluntarily’ [8]. In principle, either a woman would have to file a complaint with the police against a family member or someone would have to do so on her behalf [9, 10]. A news article published in 2019 noted that no single police complaint against chhaupadi had been filed in Achham, one of the districts in the western part of Nepal, or in other neighbouring districts (e.g., Dailekh); thus, authorities face an immense challenge in implementing the law at the local level [13]. Due to the lack of resources and the inability to negotiate health and human rights, rural women are unlikely to report cases related to the practice of chhaupadi, and in turn, families and communities continue imposing the practice on them [10].

### How effectiveness of interventions could be enhanced

Since the social components surrounding a traditional practice constitute different layers of factors in relation to individuals, groups, and communities, multilevel, multisectoral interventions are more effective than single-component interventions in changing harmful traditional practices and producing individual-level outcomes [21, 22]. One of the examples of such multisectoral approaches could be a rights-based strategy including interventions focused on supporting social justice, human rights, and community development; promoting literacy and empowerment among young girls and women; and reducing overall gender discrimination [20]. Moreover, incorporating menstrual health and hygiene into comprehensive water, sanitation and hygiene interventions could include activities such as community meetings, interaction programmes, workshops, and awareness-raising activities that could help families realize the importance of menstrual health and hygiene to young girls and women [12].

Legal approaches against chhaupadi will only be effective if they can create an enabling environment by involving different stakeholders across different levels [10]. For instance, a proper mechanism for a young girl or woman to file a complaint against her family members at the local level (e.g., schools, neighbourhood or local support groups) for imposing harmful customs should be in place. However, this is only possible through the coordination between the police and local community groups, such as schools, children’s clubs, local women’s groups, mothers’ groups and local organizations [9].

Most importantly, community power relations, perceptions and experiences should not be ignored because they function as a means by which to understand the influence of community-based values, perceived threats, stigma, rejection, and the social pressure to reinforce the practice of menstrual traditions by women and families [5]. These perceptions reflect community attitudes of non-acceptance of interventions that are thought to be driven by an external Western ideals that undermine and ignore community-based indigenous and traditional practices [19]. Interventions that aim to change harmful traditions such as the menstrual taboo need to consider the integral parts of the socio-cultural context such as menstruation-related myths, stigma and denial. However, this is only possible if the interventions work with—but not against—the prevailing cultural practices and beliefs*.*

Interventions that incorporate community mobilization components, in which the community members, the traditional or religious leaders, community health volunteers or local women’s groups are mobilized to implement the interventions, are likely to be more responsive to the local context and cultural groups [17]. Emphasis should be placed on including elements of community dialogue, understanding the importance of local rewards and punishments, and facilitating change among the social groups that include men and women from multiple generations within the community [20]. However, it is very difficult to evaluate and identify which component/s of the proposed complex multilevel, multisectoral intervention/s would be more effective than others; therefore, interdisciplinary research that combines social science and public health approaches to investigate the contexts, mechanisms and outcomes of such interventions is recommended [18].

## Conclusions

In this article, we have noted that menstrual management interventions, including activities to eliminate chhaupadi, are only effective for a short duration of time. The reasons identified include (a) poor involvement of key community stakeholders during the implementation of such interventions, (b) stigma and acts of denial towards women who stop practising menstrual traditions, (c) influence of contextual factors (e.g., low income and illiteracy) on such interventions, (d) resistance from community leaders and traditional healers, and (e) the poor implementation of laws against chhaupadi.

Multilevel, multisectoral interventions (e.g., interventions supporting social justice; human rights, and community development; promoting literacy and empowerment among young girls and women; programmes promoting menstrual health and hygiene; awareness-raising activities) are more effective than single-component interventions in changing harmful traditional practices and producing individual-level outcomes. In addition, interventions that have an active community mobilization component could be more responsive to local contexts and cultural groups.

## Data Availability

Not applicable.
